# Tailoring and evaluating an intervention to improve shared decision-making among seniors with dementia, their caregivers, and healthcare providers: study protocol for a randomized controlled trial

**DOI:** 10.1186/s13063-018-2697-1

**Published:** 2018-06-25

**Authors:** Anik M. C. Giguere, Moulikatou Adouni Lawani, Émilie Fortier-Brochu, Pierre-Hugues Carmichael, France Légaré, Edeltraut Kröger, Holly O. Witteman, Philippe Voyer, Danielle Caron, Charo Rodríguez

**Affiliations:** 1 Department of Family Medicine and Emergency Medicine, Laval University, Pavillon Ferdinand-Vandry, room 2881-C, 1050 avenue de la Médecine, Quebec, QC G1V 0A6 Canada; 2 Quebec Centre for Excellence on Aging, St-Sacrement Hospital, Room L2-21, 1050, chemin Sainte-Foy, Quebec City, Quebec Canada; 3 Laval University Research Centre on Primary Care and Services, Quebec City, Quebec Canada; 4 Research Axis of Population Health and Practice-Changing Research Group, CHU de Quebec Research Centre, Quebec city, QC Canada; 5 Faculty of Nursing Sciences, Laval University, Pavillon Ferdinand-Vandry, room 2881-C, 1050, avenue de la Médecine, Quebec, QC G1V 0A6 Canada; 6 Faculty of Pharmacy, Laval University, St-Sacrement Hospital, Room L2-30, 1050, Chemin Sainte-Foy, Québec, QC G1S 4L8 Canada; 7 Department of Family Medicine, Faculty of Medicine, McGill University, 5858 chemin de la Cote-des-Neiges, 3rd floor, Suite 300, Room 328, Montreal, Quebec Canada

**Keywords:** E-learning, Evidence summary, Knowledge translation, Clinical tool, Caregiver, Alzheimer, Aging, Primary care, Patient partnership, Aging

## Abstract

**Background:**

The increasing prevalence of Alzheimer’s disease and other forms of dementia raises new challenges to ensure that healthcare decisions are informed by research evidence and reflect what is important for seniors and their caregivers. Therefore, we aim to evaluate a tailored intervention to help healthcare providers empower seniors and their caregivers in making health-related decisions.

**Methods:**

In two phases, we will: (1) design and tailor the intervention; and (2) implement and evaluate it. We will use theory and user-centered design to tailor an intervention comprising a distance professional training program on shared decision-making and five shared decision-making tools dealing with difficult decisions often faced by seniors with dementia and their caregivers. Each tool will be designed in two versions, one for clinicians and one for patients. We will recruit 49 clinicians and 27 senior/caregiver to participate in three cycles of design-evaluation-feedback of each intervention components. Besides think-aloud and interview approaches, users will also complete questionnaires based on the Theory of Planned Behavior to identify the factors most likely to influence their adoption of shared decision-making after exposure to the intervention. We will then modify the intervention by adding/enhancing behavior-change techniques targeting these factors. We will evaluate the effectiveness of this tailored intervention before/after implementation, in a two-armed, clustered randomized trial. We will enroll a convenience sample of six primary care clinics (unit of randomization) in the province of Quebec and recruit the clinicians who practice there (mostly family physicians, nurses, and social workers). These clinics will then be randomized to immediate exposure to the intervention or delayed exposure. Overall, we will recruit 180 seniors with dementia, their caregivers, and their healthcare providers. We will evaluate the impact of the intervention on patient involvement in the decision-making process, decisional comfort, patient and caregiver personal empowerment in relation to their own healthcare, patient quality of life, caregiver burden, and decisional regret.

**Discussion:**

The intervention will empower patients and their caregivers in their healthcare, by fostering their participation as partners during the decision-making process and by ensuring they make informed decisions congruent with their values and priorities.

**Trial registration:**

ClinicalTrials.org, NCT02956694. Registered on 31 October 2016.

**Electronic supplementary material:**

The online version of this article (10.1186/s13063-018-2697-1) contains supplementary material, which is available to authorized users.

## Background

In 2016, 564,000 Canadians aged 65 years and over were living with dementia; it is estimated that this number will increase to 937,000 by 2031 [1]. The medications available to treat dementia are of limited efficacy and can cause important side effects [2, 3]. Non-pharmacological alternatives may help with some symptoms, but patients, their caregivers, and their primary healthcare providers are less familiar with their benefits and harms [4]. In such clinical situations, the shared decision-making model proposes that clinicians and patients collaborate to make joint decisions based on the best evidence on benefits and harms of all available health options (including watchful waiting), and on patient values and preferences in regard to those options [5].

Innovative strategies are thus needed to ensure that decisions about healthcare options for seniors living with dementia are informed by the best scientific evidence and take into account the patients’ circumstances and preferences [6]. Clinicians should learn to communicate effectively scientific information, ensure patient understanding, and identify patient preferences [7–9]. For people with dementia and other frail patients, healthcare goals are more often directed toward improving wellbeing than toward cure or increased survival [10–12]. Clinicians should thus also learn to identify patient and caregiver priorities for managing functional status and the identification of healthcare goals and end-of-life preferences [12, 13].

Training of health professionals can facilitate shared decision-making [14–16], especially if training incorporates the use of patient decision aids [17]. We have thus developed decision boxes (DBs), that help clinicians and patients weigh the benefits and harms of health options in light of what matters to patients. To ensure that the DBs meet the needs of their specific audiences, we have developed two versions: one designed for clinicians and the other, a simplified version, for patients/caregivers. Our previous work showed that DBs are valued by clinicians and patients [18, 19]. However, several unaccounted-for factors might limit communication and shared decision-making with seniors living with dementia, comprising literacy issues [20], the involvement of caregivers in the decision-making process [21, 22], sensory deficits (deafness, visual acuity), a greater propensity of elders to rely on health professionals for their decision-making [23], and cognitive deficits. It is thus essential to tailor the earlier DB model to seniors living with dementia and their caregivers, to address some of these challenges, as our previous results suggest that DBs designed with feedback from users are better adapted to their needs [24].

Professional training and research on shared decision-making in the context of dementia are still lacking. Recent research described the processes, challenges, and trajectories of shared decision-making in dementia care [25–27]; one study reported an approach to develop a computer-based decision aid for seniors with dementia [28, 29]. To the best of our knowledge, a single study developed and evaluated a decision aid intervention, in the context of dementia, for people with advanced dementia who lived in nursing homes [30]. As primary care clinicians play a central role in providing care to this population [31], it is now a priority to train them and develop patient decision aids that will meet their important training needs caring for this population [32].

Therefore, in a recent survey, we identified five difficult decisions that patients living with dementia and their informal caregivers often face in primary care settings [33]: (1) choosing a support option to reduce the burden of informal caregivers or to improve their quality of life; (2) choosing a treatment to manage agitation, aggression, or psychotic symptoms (3) deciding whether to stop driving following diagnosis (4) choosing an option to ensure quality of life and comfort and (5) deciding whether of not to prepare advance directives.

**Fig. 1:**
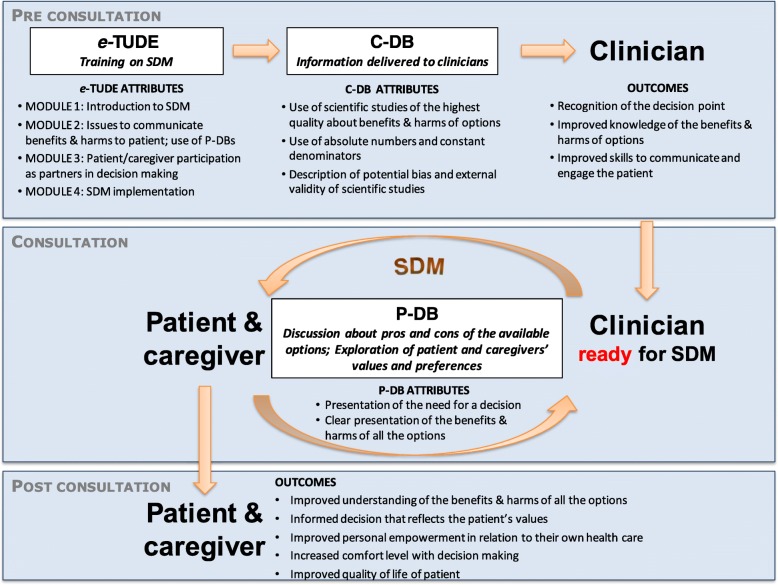
Logic model of the multicomponent intervention, comprising the Clinician-Decision Box (C-DB), the Patient-Decision Box (P-DB), and the professional training program e-TUDE, with the mechanisms by which they support shared decision-making and their impact, inspired from the conceptual models of the Decision Box [24] and DECISION+ [67]

In the current study, we thus propose to tailor and evaluate an e-learning professional training program on shared decision-making, comprising DBs on the health options to consider before making these five difficult decisions. A pilot study already confirmed the feasibility of clinician recruitment rates (63%) and questionnaire reponse rates (61%) and the acceptability of the intervention [34]. We also already adapted our implementation strategy to limit barriers to adopting the DBs in primary care practice that we identified in a previous study [24, 35]. We expect that the tailored intervention will improve shared decision-making between clinicians and patients with dementia and their caregivers, and in turn improve patient and caregiver empowerment in relation to their own healthcare (Fig. 1).

## Methods/Design

### Study design

The study will be conducted in two phases, using interdisciplinary approaches from knowledge translation science, cognitive psychology, and usability engineering, with a goal of designing a theory-based knowledge translation intervention to implement shared decision-making. Our specific objectives are, first, to involve users in the iterative design of each component of the intervention and, second, to implement and evaluate the effectiveness of the intervention using a clustered randomized controlled trial (RCT) design.

### Study participants and recruitment strategy

We will recruit convenience samples of primary healthcare providers who work in family medicine clinics (mostly family physicians, nurses, and social workers), their senior patients (aged 65+ years) diagnosed with dementia, and the patients’ informal caregivers. To begin with, we will email the directors of the family medicine clinics in the province of Quebec (Canada) to ask if we can recruit clinicians in their clinics. When they agree, we will present the study during a regularly scheduled meeting. After the presentation, clinicians in attendance will be invited to participate in the study and those who accept will complete the study entry questionnaire.

We will request that clinicians who agree to participate ask their senior patients (65+ years) with dementia and their informal caregivers, if they have one, permission to be contacted by a member of the research team to solicit their participation. The research team will meet those who accept to complete study questionnaires before their consultation with participating physicians.

From the pool of clinics that agree to participate, we will select a convenience sample of six clinics located nearest to our research center (Quebec City) to participate in the RCT. We will then select a second convenience sample of three clinics that are the next nearest to Quebec City to participate in the user-centered designs of both the e-TUDE e-learning program and the patient DBs. A final convenience sample of five clinics located elsewhere in the Province of Quebec will then be selected to participate in the user-centered design of clinician DBs. Each person will only be invited to one of these studies.

### Phase 1: user-centered design of each component of the intervention

In phase 1, we will use user-centered approaches to design: (1) five DB prototypes for clinicians (C-DB); (2) five DB prototypes for patients and their informal caregivers (P-DB); and (3) a 70-min e-learning professional program on shared decision-making (e-TUDE).

#### Prototype development

e-TUDE will include four modules, at the end of which the clinician will be able to: (1) explain SDM, its foundations, its advantages, and disadvantages; (2) use best practices to communicate risks and uncertainty and allow patients to understand the issues involved in their decisions; (3) use various strategies, including the DBs, to identify the values and preferences of patients; and (4) integrate all learnings to engage the patient and caregiver in a shared decision-making process. Guided by another professional training program that has been developed and assessed by our team [14], it will include narrated slides, videos, interactive quizzes, and exercises. Several content experts (in shared decision-making, medical education, and instructional design) reviewed the training program before user testing.

DBs cover health questions that have no single best answer and are framed to help weigh the benefits/harms of all options in light of the patient’s individual health status, as previously published [19, 24]. To adapt the tool to the needs of each type of user, the DB includes a version designed for clinicians (C-DB) and a simplified version for patients/caregivers (P-DB). The C-DB is designed as a continuing education activity. It provides clinicians scientific information to carefully review before their consultations with patients. It is more succinct than the P-DB and allows the clinician to critically appraise the evidence by describing the design and participants of included studies and synthesizing study limitations using the GRADE approach [36]. The P-DB is designed to be used during the consultation to encourage discussion and to be left with the patient and caregiver to review after the consultation. It is distinct from the C-DB, as it presents the information in complete sentences, uses larger font sizes, and comprises a value clarification exercise and an instrument to screen for decisional conflict. We will design the P-DB guided by the Ottawa Decision Support Framework [37, 38] and current international standards for decision aids [39]. Several clinical experts involved in the care of seniors living with dementia (healthcare professionals, informal caregivers, managers, representatives of community-based organizations devoted to these seniors, and clinical researchers involved in the organization of primary care or services delivered to seniors with dementia in the Province of Quebec) reviewed the prototypes before formal testing.

#### User-centered and theory-based tailoring

##### Procedure

**Fig. 2:**
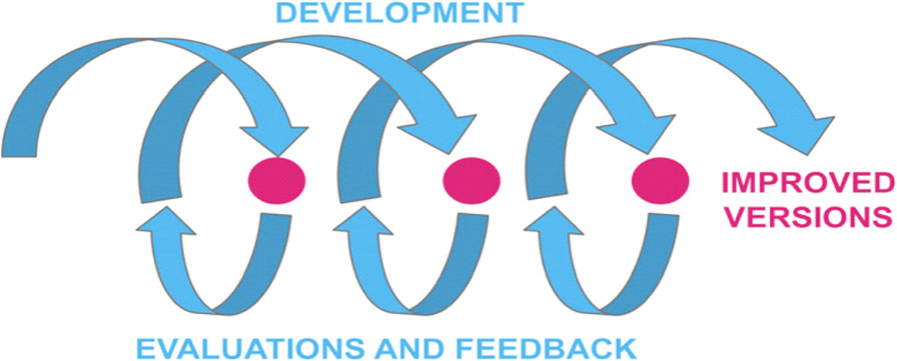
Representation of the user-centered design, which consists in alternative cycles of development and evaluation/feedback. Reproduced from Rosenbaum [41] with permission from Sarah Rosenbaum

Using a user-centered design approach [40–42], we will invite users to evaluate the usability of each component of the intervention through questionnaires and interviews (Table 2). We will ask them to complete a questionnaire based on the Theory of Planned Behavior (TPB) [43, 44] to identify the factors that are likely to limit their adoption of shared decision-making (along a number of domains namely, intention, social influence, beliefs about capabilities, moral norm, attitude/beliefs about consequences). If on a scale of 1–7, with 1 being the lowest, participant mean ratings fall < 4 for a given factor (4 being the middle of the scale), then we will modify the intervention by adding/enhancing behavior-change techniques targeting this lower factor, inspired from an approach described elsewhere [45, 46]. We will also improve usability based on the ratings on the other scales and think-aloud results. Results of this first stage will be followed by a new cycle of design and evaluation. This iterative process will continue until: (1) we observe indications of good usability and no significant problems; (2) users report that they intend to engage in shared decision-making; or (3) we have conducted a maximum of three rounds of design-evaluation-feedback (Fig. 2). For e-TUDE, each cycle will provide feedback from five users, for a total of 15 user evaluations. For the C-DB, each cycle will provide feedback from six users, for a total of 18 user evaluations per tool. For the P-DB, each cycle will provide feedback from three patient-caregiver groups, giving a total of nine evaluations per P-DB. These numbers respect human factor validation testing [47].

##### e-TUDE tailoring

A total of 15 clinicians will be invited to review the tutorial during a think-aloud session conducted at their clinic. Initially, a first series of five clinicians will complete e-TUDE in the presence of a trained moderator who will ask some questions from a semi-structured interview guide, noting any need for technical support or assistance in a logbook. When they will have completed e-TUDE, the moderator will ask them to complete a questionnaire to identify the factors that are likely to limit their adoption of shared decision-making using the ICanSDM scale (a novel scale based on a review of the published barriers and myths to adopting SDM, personal communication of Anik Giguere) and other measures (Table 2). We will then analyze the answers from these five clinicians and modify e-TUDE to tailor it better to their needs. Two more series of five clinicians will go through the same process, allowing to tailor the tutorial at each cycle (Table 1).

**Table 1:** Steps of the user-centered design process for (a) e-TUDE, (b) the Clinician-Decision Boxes (C-DBs), and (c) the Patient-Decision Boxes (P-DBs)

(A) e-TUDE					
Round 1 evaluation	Clinicians #1–5				
Tailoring	↓				
Round 2 evaluation	Clinicians #6–10				
Tailoring	↓				
Round 3 evaluation	Clinicians #11–15				
Tailoring and final version					
(B) Clinician-Decision box					
	C-DB 1	C-DB 2	C-DB 3	C-DB 4	C-DB 5
Round 1 evaluation	Clinicians #16–22	Clinicians #23–29	Clinicians #30–36	Clinicians #37–42	Clinicians #43–49
Tailoring	↓	↓	↓	↓	↓
Round 2 evaluation	Clinicians #43–49	Clinicians #16–22	Clinicians #23–29	Clinicians #30–36	Clinicians #37–42
Tailoring	↓	↓	↓	↓	↓
Round 3 evaluation	Clinicians #37–42	Clinicians #43–49	Clinicians #16–22	Clinicians #23–29	Clinicians #30–36
Tailoring and final version					
(C) Patient-Decision Boxes					
	P-DB 1	P-DB 2	P-DB 3		
Round 1 evaluation	Patient + CG #1–3	Patient + CG #4–6	Patient + CG #7–9		
Tailoring	↓	↓	↓		
Round 2 evaluation	Patient + CG #10–12	Patient + CG #13–15	Patient + CG #16–18		
Tailoring	↓	↓	↓		
Round 3 evaluation	Patient + CG #19–21	Patient + CG #22–24	Patient + CG #25–27		
Tailoring and final version					

*CG* caregiver

##### Clinician-decision box (C-DB) tailoring

A total of 30 clinicians will participate to the review of the five C-DBs. Each of the participating clinician will review three C-DBs, at a rate of one per month over three months, giving six clinicians per C-DB. We will send clinicians a link to one C-DB by email and the C-DB will also be accessible on our website [48]. After reviewing this first C-DB, clinicians will be invited to complete the questionnaire to identify the factors that are likely to limit their adoption of shared decision-making and other measures (Table 2). Their answers will be used to tailor the intervention, as described in the previous paragraph. After this initial evaluation and tailoring, we will send each C-DB to six different users from the same pool of 30 clinicians (Table 1). Overall, we aim to collect a total of 90 clinician assessments of the C-DBs (i.e. 6 clinicians/clinic × 5 clinics × 3 evaluations/clinician).

**Table 2:** Phase one (tailoring) data collection steps and outcomes

Step	Outcomes (measures)
e-TUDE (clinician)	
Study entry	• Self-reported sociodemographic characteristics • Self-reported interest for each of the topics addressed in the C-DB on a 1–10 visual analogue scale
Pre training	• Role preference scale [63] • Perceptions of being able to adopt shared decision-making using the ICanSDM scale • Intention to engage senior patients living with dementia and their caregivers in decision-making about choosing a health intervention, based on the TPB [37, 38]
Post training	• Satisfaction with e-TUDE (1–5 smiley face scale) • Usability of e-TUDE, based on the Technology Acceptance Model (TAM-2) [64–66] • Role preference scale [63] • Perceptions of being able to adopt shared decision making using the ICanSDM scale • Intention to engage senior patients living with dementia and their caregivers in decision-making about choosing a health intervention, based on the TPB [37, 38]
C-DB (clinician questionnaire)	
Study entry questionnaire	• Self-reported sociodemographic characteristics • Self-reported interest for each of the topics addressed in the C-DB on a 1–10 visual analogue scale
After reviewing each C-DB	• Satisfaction with C-DB (1–5 smiley face scale) • C-DB usability based on the *Technology Acceptance Model* (TAM-2) [64–66] • Intention to use what was learned from the C-DB to explain to patients the benefits and harms of the options, based on the TPB [37, 38] • Value of the evidence presented in the C-DB, measured using the clinician version of the IAM [43, 67]
P-DB (Patients and caregivers)	
Able patient	• Socio-demographic characteristics of the patient self-reported by the patient • Satisfaction with the P-DB (1–5 smiley face scale) • P-DB usability based on the *Technology Acceptance Model* (TAM-2) [64–66] • Value of the evidence presented in the P-DB, measured using the patient version of the IAM [67]
Caregiver of able patient	• Sociodemographic characteristics of the caregiver: self-reported by the caregiver • Satisfaction with the P-DB (1–5 smiley face scale) • P-DB usability based on the *Technology Acceptance Model* (TAM-2) [64–66] • Value of the evidence presented in the P-DB, measured using the caregiver version of the IAM [67]
Caregiver of unable patient	• Self-reported sociodemographic characteristics of the caregiver • Sociodemographic characteristics of the patient reported by the caregiver • Satisfaction with the P-DB (1–5 smiley face scale) • P-DB usability based on the *Technology Acceptance Model* (TAM-2) [64–66] • Value of the evidence presented in the P-DB, measured using the caregiver version of the IAM [67]

##### Patient-decision box (P-DB) tailoring

Because of recruitment challenges in this vulnerable population, we assess only three of the five P-DBs developed. We will then apply our findings to format all P-DBs, including those which have not been evaluated. Patients and/or their caregiver will be invited to review a P-DB together during a semi-structured interview and then to complete questionnaires. To ensure assessment of all the P-DBs, we randomly assigned a P-DB to each patient/caregiver dyad. The objectives of the 45-min interview will be to: (1) identify the decision-making needs of participants relative to the decision presented in the P-DB; (2) distinguish the reasons influencing their decisions relative to the clinical situation presented; and (3) solicit feedback on changes needed to improve the usability of the P-DB. After the interview, participants will be invited to complete questionnaires to identify the factors that are likely to limit their adoption of shared decision-making and other measures (Table 2) to inform the ongoing design of the intervention, as described earlier. We will obtain feedback from three patient–caregiver groups before modifying the P-DB and asking another group (total of 27 patient–caregiver groups: recruited at a rate of 9 groups/clinic/month × 3 clinics during one month) (Table 1).

##### Data analysis

We will transcribe verbatim all the audio-recorded discussions. Transcripts and comments collected in the free-text fields of the questionnaires will be imported as source documents into N’Vivo. To begin with, two coders (a research assistant and a student) will independently conduct a thematic content analysis of the proposed changes and problems outlined during the think-aloud evaluation of e-TUDE and in answers to open-ended questions of the Information Assessment Method (IAM) for the C-DB. Following this preliminary analysis, the coders, the principal investigators, and a co-investigator with expertise in human factors (HOW) will discuss the functionality of each tool, agree on modifications to improve functionality and modify the prototypes accordingly.

Using data from the questionnaires, we will evaluate whether user experience and intention to adopt shared decision-making change significantly between rounds.

To ensure the relevance and consistency of coding, the principal investigator (AG) will randomly check the analysis (10% of interviews) at three different times.

### Phase 2: implementation and evaluation of the intervention

#### Study design

**Fig. 3:**
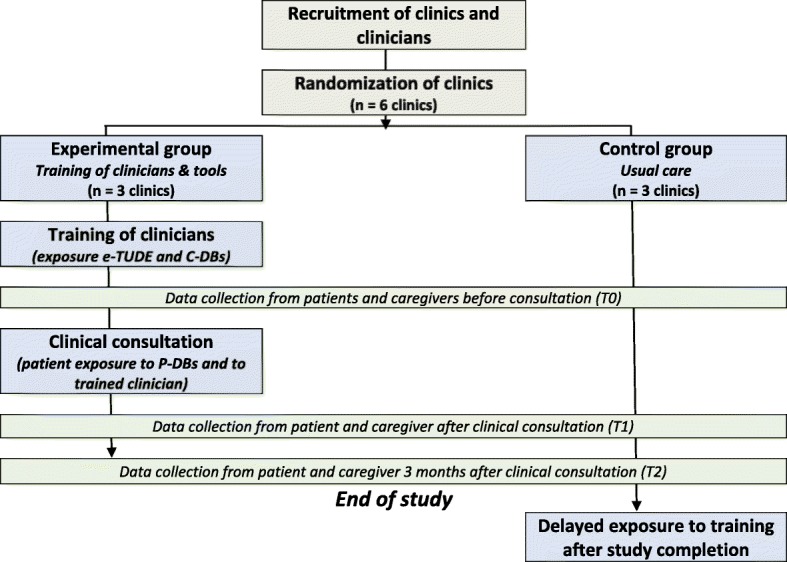
Clustered randomized trial design

In this second phase, we will implement the intervention and evaluate its impact. We will use a two-armed, superiority, parallel-group, clustered RCT with a pre-post evaluation of the impact of the program (Fig. 3). We randomized family medicine clinics to minimize contamination. A biostatistician who is not involved in the main research will use a computer random number generator to randomize the clinics to: (1) immediate exposure to the training program (experimental group); or (2) usual care with delayed exposure to the program (control group). We will monitor implementation throughout the study and will tailor implementation if needed. The protocol of the trial described in this second phase addresses recommendations from the SPIRIT 2013 © checklist (Additional file 1).

#### Experimental group (training of clinicians and tools)

In the clinics allocated to the experimental group (immediate training), the clinicians who agree to participate will have access to e-TUDE for one month to complete it. Thereafter, they will receive the five C-DBs by email, at a rate of one per week for five weeks. Upon receipt of each C-DB, participants will be asked to complete the Information Assessment Measure [49]. Furthermore, each participating clinician will also be invited to use the five P-DBs with their patients. To this end, they will receive a stack of printed copies of the P-DBs for use in home care consultations and we will also leave some copies in a wall file in each consultation room of the clinic.

We will evaluate intervention fidelity using a process measure, the OPTION scale [50–52]. Based on audio-recordings of the clinical consultation, two trained observers will rate the clinicians’ level of expertise for 12 key “patient-involving” behaviors. In case of any disagreement, the observers will discuss to reach consensus.

#### Control group (usual care)

Clinicians from the clinics allocated to the control group (delayed training) will practice as usual and have access to the same training program, but after the end of data collection (Fig. 3).

#### Data collection

Clinicians from the clinics allocated to the experimental group will complete questionnaires after their training with e-TUDE and after receiving each C-DB (Table 3). These data will allow us to evaluate whether the intervention changes clinicians’ perceptions of being able to adopt shared decision-making using the novel ICanSDM scale (Anik Giguere, personal communication), the role they prefer during the clinical decision-making process, and their intention to engage senior patients living with dementia and their caregivers in decision-making about a choice of health options.

**Table 3:** Phase two (RCT) data collection steps, outcomes, processes measures, and confounding variables

Step	Outcomes (measures)
Clinician baseline, at recruitment (t0)	
Clinician, at recruitment	• Self-reported sociodemographic characteristics (confounding variables) • Self-reported interest for each of the topics addressed in the C-DB on a 1–10 visual analogue scale
Clinician, during professional training (only for those allocated to the experimental group)	
Before e-TUDE	• Role preference scale [63] • Perceptions of being able to adopt shared decision-making using the ICanSDM scale • Intention to engage senior patients living with dementia and their caregivers in decision-making about choosing a health intervention, based on the TPB [37, 38]
After e-TUDE	• Satisfaction with e-TUDE (1–5 smiley face scale) • e-TUDE usability based on the *Technology Acceptance Model* (TAM-2) [64–66]
After reception of each C-DB	• Satisfaction with DB (1–5 smiley face scale) • DB usability based on the *Technology Acceptance Model* (TAM-2) [64–66] • Value of the evidence presented in the P-DB, measured using the clinician version of the IAM [43, 67]
When training is completed	• Role preference scale [63] • Perceptions of being able to adopt shared decision-making using the ICanSDM scale • Intention to engage senior patients living with dementia and their caregivers in decision-making about choosing a health intervention, based on the TPB [37, 38]
Patient baseline, before clinical consultation (t0)	
Able patient	• Sociodemographic characteristics of the patient: self-reported by the patient (confounding variable) • Patient empowerment using the Healthcare empowerment questionnaire [47] (patient PRIMARY outcome) • Patient quality of life using the French-validated QoL-AD [50, 51] (patient outcome)
Caregiver of able patient	• Self-reported sociodemographic characteristics of the caregiver (confounding variable) • Caregiver empowerment using the Healthcare empowerment questionnaire [47] (caregiver outcome) • Caregiver burden [48, 49] (caregiver outcome) • Patient quality of life using QoL-AD [50, 51] (patient outcome)
Caregiver (and legal representative) of unable patient	• Self-reported sociodemographic characteristics of the caregiver (confounding variable). • Sociodemographic characteristics of the patient (confounding variable) • Caregiver empowerment using the Healthcare empowerment questionnaire [47] (caregiver outcome) • Caregiver burden [48, 49] (caregiver outcome) • Patient quality of life using QoL-AD [50, 51] (patient outcome)
During consultation	
Audio-recordings of the clinical consultations (OPTION-12)	• Patient involvement in decision-making, using the third-observer OPTION-12 scale [44–46] (process measure)
After clinical consultation (t1)	
Able patient	• Self-reported comfort-level with decision-making measured with the Decisional conflict scale [54, 55] (patient PRIMARY outcome) • Self-reported perceptions of the decision-making processes using the 3-item Collaborate instrument [52, 53] (process measure) • Questions about the clinical visit
Caregiver of able patient	• Self-reported comfort-level with decision-making measured with the Decisional conflict scale [54, 55] (caregiver outcome) • Self-reported perceptions of the decision-making processes using the 3-item Collaborate instrument [52, 53] (process measure) • Questions about the clinical visit
Caregiver (and legal representative) of unable patient	• Self-reported comfort-level with decision-making measured with the Decisional conflict scale [54, 55] (caregiver outcome) • Self-reported perceptions of the decision-making processes using the 3-item Collaborate instrument [52, 53] (process measure) • Questions about the clinical visit
Six months after clinical consultation (t2)	
Able patient	• Patient empowerment using the Healthcare empowerment questionnaire [47] (patient PRIMARY outcome) • Patient quality of life using the QoL-AD [50, 51] (patient outcome)
Caregiver of able patient	• Caregiver burden using a French-validated questionnaire [48, 49] (caregiver outcome) • Decisional regret [56, 57] • Patient quality of life as perceived by the caregiver QoL-AD [50, 51] (patient outcome) • Caregiver empowerment using the Healthcare empowerment questionnaire [47] (patient PRIMARY outcome)
Caregiver (and legal representative) of unable patient	• Caregiver burden using a French-validated questionnaire [48, 49] (caregiver outcome) • Decisional regret [56, 57] • Patient quality of life as perceived by the caregiver using the QoL-AD [50, 51] (patient outcome) • Caregiver empowerment using the Healthcare empowerment questionnaire [47] (patient PRIMARY outcome)

*C-DB* clinician-decision box, *P-DB* patient-decision box

After the clinicians in the experimental study group have been exposed to e-TUDE and the C-DBs, the research team will start data collection in both the control and experimental study groups. We will meet the patients/caregivers who accepted to participate at the clinic, immediately before their next consultation. They will complete the study entry questionnaires and we will assess baseline levels of patient and caregiver empowerment in healthcare (the primary outcome) [53]. We will also assess caregiver burden [54, 55] as well as patient quality of life using the QoL-AD questionnaire that will be completed by the patient, if s/he is able to do so, and by the caregiver [56, 57].

Then, participating clinicians will audio-record the discussion during the consultation and the recording will be transcribed verbatim by a professional transcriber. This will allow measuring patient/caregiver involvement in the decision-making process using the third-observer OPTION-5 scale.

Right after the consultation, we will ask patients and caregivers their perceptions of the decision-making process during the consultation using the three-item CollaboRATE instrument [58, 59] and their comfort-level with decision-making using the Decisional Conflict Scale [60, 61]. Three months after consultation, we will meet patients and caregivers at their home to ask them to complete again a questionnaire with questions about healthcare empowerment, caregiver burden [54, 55], patient quality of life [56, 57], and decisional regret [62, 63].

All data will be entered electronically in double by two research assistants, at the coordinating center (Laval University). Checks will be applied at the time of data entry into a specific field, before the data are written to the database. Data integrity will be enforced through a variety of mechanisms: referential data rules; valid values; range checks; and consistency checks against data already stored in the database. The option to choose a value from a list of valid codes and a description of what each code means will be available where applicable.

#### Power calculation

We estimate that a sample of 162 patients will allow us to detect a mean difference of 1.476 in their empowerment in healthcare (corresponding to an effect size of 0.6) between our two groups. Assuming a standard deviation of 2.46 and an ICC (intraclass correlation coefficient) of 0.02, such a sample would give us a statistical power of 88% to detect the proposed mean difference at a significance level of 5% (Table 4). To account for an attrition and missing data rate of 10%, we have set a target of 180 patients. A previous study conducted by our research team yielded an ICC of 0.02 in a similar setting when measuring the frequency with which clinicians prescribed antibiotics after training in SDM [64]. We estimate that this convenient sample of 162 patients post intervention will allow us to detect a mean difference of 2.6% (SD = 4.8, Cohen’s D = 0.55) in decisional comfort (Decisional Conflict Scale) considering an ICC of 0.02 at a significance level of 0.05 and with a power of 80%.

**Table 4:** Effect of study length on the power of the study

Scenario	Estimated power	Threshold of statistical significance	HCEQ Standard Deviation	Minimum detectable difference (Cohen’s effect size of 0.6)	Intra-cluster correlation	Adjusted standard deviation	Number of clusters (clinics)	Totalsample size	Study length (months)	Mean number of patients per clinic
1	0.519	0,05	2.46	1.476	0,02	2.65	6	54	1	9
2	0.76	0,05	2.46	1.476	0,02	2.85	6	108	2	18
3	0.88	0,05	2.46	1.476	0,02	2.98	6	162	3	27
4	0.92	0,05	2.46	1.476	0,02	3.21	6	216	4	36
5	0.948	0,05	2.46	1.476	0,02	3.37	6	270	5	45
6	0.963	0,05	2.46	1.476	0,02	3.53	6	324	6	54

Despite a relatively small number of clusters (six clinics to be randomized), we are confident that practice variation will be low enough to allow similar groups at baseline. Indeed, a recent systematic literature review reports rather uniform and low adoption of shared decision-making in the general practices in Canada, with two studies reporting OPTION scores of 24 ± 8 on a scale of 0–100 for one study (*n* = 152) and 19 ± 7 (*n* = 41) for the other.

#### Data analysis

We will evaluate response rates as the number of clinics recruited divided by the number invited and the number of clinicians who agree to participate in the study divided by the number invited. We will also calculate patient recruitment rates as the number of patients recruited per attending physician per day. Audio-recordings of consultations will be collected by the research team at the end of each day and transcribed verbatim. Two trained research assistants will analyze the transcripts independently using the OPTION-5 scale [50–52] to quantify patient/caregiver involvement in the decision-making process during consultations.

We will perform descriptive statistical analyses of all the outcomes and measures. To compare the two study arms on primary and secondary outcomes, we will use hierarchical models to take into account the clustering effects of clinics and clinicians, intervention version, and repeated measures (for outcomes measured three times or more). For clinician-based outcomes, the analysis will take into account the decision considered, clinical site, sociodemographic characteristics of clinicians, and rural/urban localization of the clinic. For patient-based outcomes, the analyses will take into account clinical site, sociodemographic characteristics and dementia severity of patients, and rural/urban localization of the clinic. We will analyze all data following intention-to-treat principles. Quantitative data will be analyzed using the SAS statistical package (version 9.4).

To verify intervention fidelity, we will perform a concurrent analysis of the implementation processes (patient involvement in decision-making).

For each variable analyzed, according to the type of variable (continuous or categorical), the goodness of fit and the assumptions of each model will be assessed. We will analyze all data following intention-to-treat principles. Additionally, if the number of missing data is relatively low, then, in addition to analyzing the data by excluding missing observations, we will also perform a multiple imputation procedure on the full model and verify that results were not affected by missing information.

## Discussion

By designing tools that help healthcare professionals empower seniors to make clinical decisions on issues that affect their own health, this initiative supports seniors’ wellbeing and independence, the latter being one of the four principles of healthy aging [65]. The training program will provide clinicians, patients living with dementia, and their caregivers access to best available evidence on health options, so that together they can decide on next steps. In their role as partners in decision-making, seniors and their caregivers will experience more decisional comfort and have higher levels of perceived healthcare empowerment, while allowing identification of their priorities. Ultimately, this training program will ensure that patients and caregivers make informed decisions that reflect their care goals and values.

The self-learning format ensures accessibility in all regions with online access, even the most remote. The scientific information will be made available broadly to all stakeholders: physicians and residents; nurses and other health or social services professionals; patients and their caregivers. This continuity of information among the various stakeholders will ensure better coordination of care and services between the parties, resulting in better monitoring of care and support of seniors with cognitive impairment. We expect that these tools will become an essential reference for the training of health professionals across the province of Quebec and for patients with cognitive impairment and their caregivers.

Ultimately, this project will meet the training needs of clinicians who care for seniors with cognitive impairment in Quebec. Indeed, our partners and users associated with continuing professional development (CPD) have committed to implement, disseminate and build on project deliverables. Specifically, the CPD sector of the Office of Education and Continuing Professional Development of the Faculty of Medicine at Laval University will offer our e-learning training program to health professionals of every primary care organization in Quebec. We will also draw on our excellent relations and involvement in the network of the 12 teaching family medicine units of the (RUIS-Laval) and in the frontline research Network, Réseau-1 to raise awareness of the tools to users. The revenues generated through the credits for CPD course will allow to sustain the training program, notably to update the DBs and expand the program to offer DBs on additional topics.

Patient recruitment is critical in this project. Our experience in recruiting clinicians in primary care settings ensures that we will complete this important step. The delayed intervention allows that all recruited clinicians are exposed to the training program, thus facilitating recruitment. In addition, seniors living with dementia are typically underdiagnosed in primary care. Consequently, we will collaborate with the Quebec Alzheimer Plan, an implementation initiative currently underway in the Province [66], which aims to improve the identification of patients living with dementia in primary care settings. This partnership will allow targeting clinicians already trained in the identification of seniors living with dementia and a more rapid identification of key stakeholders to help with recruitment.

### Strengths and limitations

Our study of a user-centered approach will mainly rely on qualitative evaluations, which will provide rich findings to draw some first conclusions on how the approach influences the vulnerable patients’ and their caregivers’ experiences of the DBs. The quantitative results will allow preliminary conclusions on the extent to which the approaches improve implementation, due to a limited sample size that could prevent statistical significance. This study is also limited because we chose to assign tools to patient/caregiver dyads randomly, to ensure an equal number of evaluation for each tool. Consequently, the tools that participants will get might not be those they would have preferred to answer their current questions, and so this deviates from daily practice.

The pragmatic clustered RCT is the best design to draw conclusions on the impact of a complex intervention on patient-important outcomes. However, due to its complex nature, several aspects of the intervention will be challenging to control. We cannot control if the clinicians will complete the distance training program and offer the DBs to their patients, but the user-centered development improves the odds that these tools will be adopted in practice. We do not either control if the participants will want to discuss the decisions covered in the studied DB during the consultations, but the fact that some of these decisions can be discussed by participants at any stage of the disease, and that they are typically rarely discussed (e.g. choosing a support option for the caregiver, preparing advance directives and a protection mandate), will encourage their use.

### Recruitment status

**Fig. 4:**
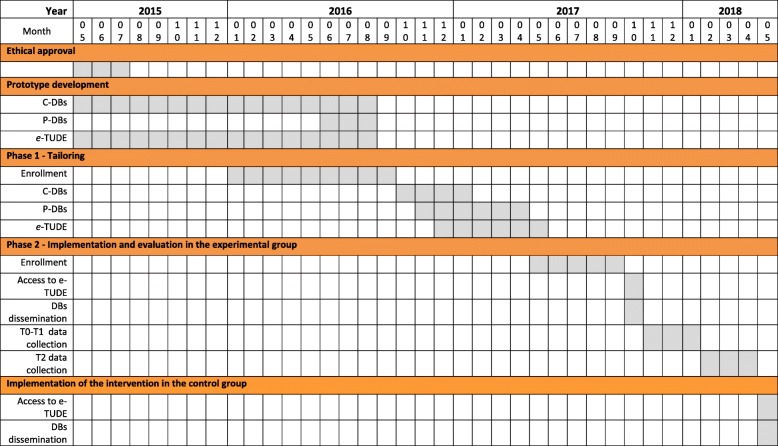
Project timeline

 Recruitement is completed. We are currently collecting data for Phase two (Fig. 4).

## Additional file

Additional file 1: SPIRIT 2013 Checklist with the items addressed in the clinical trial protocol and related documents*. (DOC 137 kb)

## Abbreviations

BPSD: Behavioral and Psychological Symptoms of Dementia
C-DB: Clinician Decision Box
CPD: Continuing professional development
IAM: Information Assessment Method
ID: Identification
P-DB: Patient-Decision Box
RCT: Randomized controlled trial
SDM: Shared decision-making
TAM: Technology Acceptance Model
TPB: Theory of Planned Behavior
