# Supplementary material for: Tailoring and evaluating an intervention to improve shared decision-making among seniors with dementia, their caregivers, and healthcare providers: study protocol for a randomized controlled trial
Source: Trials. 2018 Jun 25;19:332. doi: 10.1186/s13063-018-2697-1 (PMC6019313; doi:10.1186/s13063-018-2697-1)
Supplement: Supplementary file 1 — SPIRIT 2013 Checklist with the items addressed in the clinical trial protocol and related documents*. (DOC 137 kb) [file 13063_2018_2697_MOESM1_ESM.doc]

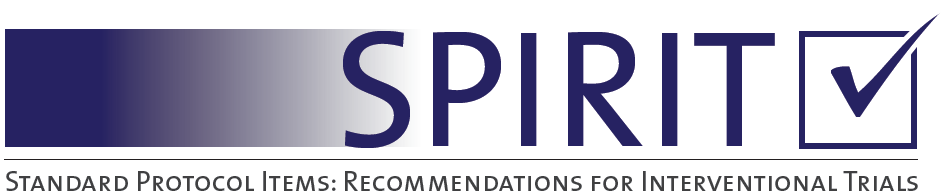


SPIRIT 2013 Checklist with the items addressed in the clinical trial protocol and related documents*

| Section/item | Item No | Description | Addressed on page number |
| --- | --- | --- | --- |
| **Administrative information** | | |  |
| Title | 1 | Descriptive title identifying the study design, population, interventions, and, if applicable, trial acronym | p1 |
| Trial registration | 2a | Trial identifier and registry name. If not yet registered, name of intended registry | **p4** |
|  | 2b | All items from the World Health Organization Trial Registration Data Set | see Appendix below |
| Protocol version | 3 | Date and version identifier | Details in the ClinicalTrials.org registry |
| Funding | 4 | Sources and types of financial, material, and other support | p21 |
| Roles and responsibilities | 5a | Names, affiliations, and roles of protocol contributors | p1, p21 |
|  | 5b | Name and contact information for the trial sponsor | Details in the ClinicalTrials.org registry |
|  | 5c | Role of study sponsor and funders, if any, in study design; collection, management, analysis, and interpretation of data; writing of the report; and the decision to submit the report for publication, including whether they will have ultimate authority over any of these activities | p21 |
|  | 5d | Composition, roles, and responsibilities of the coordinating centre, steering committee, endpoint adjudication committee, data management team, and other individuals or groups overseeing the trial, if applicable (see Item 21a for data monitoring committee) | p21 |
| Introduction |  |  |  |
| Background and rationale | 6a | Description of research question and justification for undertaking the trial, including summary of relevant studies (published and unpublished) examining benefits and harms for each intervention | p5-7 |
|  | 6b | Explanation for choice of comparators | p19 |
| Objectives | 7 | Specific objectives or hypotheses | p7 |
| Trial design | 8 | Description of trial design including type of trial (eg, parallel group, crossover, factorial, single group), allocation ratio, and framework (eg, superiority, equivalence, noninferiority, exploratory) | p13 |
| Methods: Participants, interventions, and outcomes | | |  |
| Study setting | 9 | Description of study settings (eg, community clinic, academic hospital) and list of countries where data will be collected. Reference to where list of study sites can be obtained | p8 |
| Eligibility criteria | 10 | Inclusion and exclusion criteria for participants. If applicable, eligibility criteria for study centres and individuals who will perform the interventions (eg, surgeons, psychotherapists) | p8 |
| Interventions | 11a | Interventions for each group with sufficient detail to allow replication, including how and when they will be administered | p13-14 |
|  | 11b | Criteria for discontinuing or modifying allocated interventions for a given trial participant (eg, drug dose change in response to harms, participant request, or improving/worsening disease) | NA |
|  | 11c | Strategies to improve adherence to intervention protocols, and any procedures for monitoring adherence (eg, drug tablet return, laboratory tests) | p14, 17 |
|  | 11d | Relevant concomitant care and interventions that are permitted or prohibited during the trial | NA |
| Outcomes | 12 | Primary, secondary, and other outcomes, including the specific measurement variable (eg, systolic blood pressure), analysis metric (eg, change from baseline, final value, time to event), method of aggregation (eg, median, proportion), and time point for each outcome. Explanation of the clinical relevance of chosen efficacy and harm outcomes is strongly recommended | P14-15, Tables 2-3 |
| Participant timeline | 13 | Time schedule of enrolment, interventions (including any run-ins and washouts), assessments, and visits for participants. A schematic diagram is highly recommended (see Figure) | Figure 4 |
| Sample size | 14 | Estimated number of participants needed to achieve study objectives and how it was determined, including clinical and statistical assumptions supporting any sample size calculations | p10-11, 16, Table 4 |
| Recruitment | 15 | Strategies for achieving adequate participant enrolment to reach target sample size | p19 |
| **Methods: Assignment of interventions (for controlled trials)** | | |  |
| Allocation: |  |  |  |
| Sequence generation | 16a | Method of generating the allocation sequence (e.g., computer-generated random numbers), and list of any factors for stratification. To reduce predictability of a random sequence, details of any planned restriction (eg, blocking) should be provided in a separate document that is unavailable to those who enrol participants or assign interventions | p13 |
| Allocation concealment mechanism | 16b | Mechanism of implementing the allocation sequence (e.g., central telephone; sequentially numbered, opaque, sealed envelopes), describing any steps to conceal the sequence until interventions are assigned | p13 |
| Implementation | 16c | Who will generate the allocation sequence, who will enrol participants, and who will assign participants to interventions | p13 |
| Blinding (masking) | 17a | Who will be blinded after assignment to interventions (eg, trial participants, care providers, outcome assessors, data analysts), and how | p13 |
|  | 17b | If blinded, circumstances under which unblinding is permissible, and procedure for revealing a participant’s allocated intervention during the trial | NA |
| **Methods: Data collection, management, and analysis** | | |  |
| Data collection methods | 18a | Plans for assessment and collection of outcome, baseline, and other trial data, including any related processes to promote data quality (eg, duplicate measurements, training of assessors) and a description of study instruments (eg, questionnaires, laboratory tests) along with their reliability and validity, if known. Reference to where data collection forms can be found, if not in the protocol | p14-15  Tables 2-3 |
|  | 18b | Plans to promote participant retention and complete follow-up, including list of any outcome data to be collected for participants who discontinue or deviate from intervention protocols | NA |
| Data management | 19 | Plans for data entry, coding, security, and storage, including any related processes to promote data quality (eg, double data entry; range checks for data values). Reference to where details of data management procedures can be found, if not in the protocol | p15 |
| Statistical methods | 20a | Statistical methods for analysing primary and secondary outcomes. Reference to where other details of the statistical analysis plan can be found, if not in the protocol | p16-17 |
|  | 20b | Methods for any additional analyses (eg, subgroup and adjusted analyses) | p17 |
|  | 20c | Definition of analysis population relating to protocol non-adherence (eg, as randomised analysis), and any statistical methods to handle missing data (eg, multiple imputation) | p13 |
| **Methods: Monitoring** | | |  |
| Data monitoring | 21a | Composition of data monitoring committee (DMC); summary of its role and reporting structure; statement of whether it is independent from the sponsor and competing interests; and reference to where further details about its charter can be found, if not in the protocol. Alternatively, an explanation of why a DMC is not needed | p21 |
|  | 21b | Description of any interim analyses and stopping guidelines, including who will have access to these interim results and make the final decision to terminate the trial | NA |
| Harms | 22 | Plans for collecting, assessing, reporting, and managing solicited and spontaneously reported adverse events and other unintended effects of trial interventions or trial conduct | NA |
| Auditing | 23 | Frequency and procedures for auditing trial conduct, if any, and whether the process will be independent from investigators and the sponsor | NA |
| Ethics and dissemination | | |  |
| Research ethics approval | 24 | Plans for seeking research ethics committee/institutional review board (REC/IRB) approval | p20 |
| Protocol amendments | 25 | Plans for communicating important protocol modifications (eg, changes to eligibility criteria, outcomes, analyses) to relevant parties (eg, investigators, REC/IRBs, trial participants, trial registries, journals, regulators) | p21 |
| Consent or assent | 26a | Who will obtain informed consent or assent from potential trial participants or authorised surrogates, and how (see Item 32) | p20 |
|  | 26b | Additional consent provisions for collection and use of participant data and biological specimens in ancillary studies, if applicable | NA |
| Confidentiality | 27 | How personal information about potential and enrolled participants will be collected, shared, and maintained in order to protect confidentiality before, during, and after the trial | Not in the protocol |
| Declaration of interests | 28 | Financial and other competing interests for principal investigators for the overall trial and each study site | p21 |
| Access to data | 29 | Statement of who will have access to the final trial dataset, and disclosure of contractual agreements that limit such access for investigators | NA |
| Ancillary and post-trial care | 30 | Provisions, if any, for ancillary and post-trial care, and for compensation to those who suffer harm from trial participation | NA |
| Dissemination policy | 31a | Plans for investigators and sponsor to communicate trial results to participants, healthcare professionals, the public, and other relevant groups (eg, via publication, reporting in results databases, or other data sharing arrangements), including any publication restrictions | p18-19 |
|  | 31b | Authorship eligibility guidelines and any intended use of professional writers | p21-22 |
|  | 31c | Plans, if any, for granting public access to the full protocol, participant-level dataset, and statistical code | The protocol is submitted to the Trials Journal |
| Appendices |  |  |  |
| Informed consent materials | 32 | Model consent form and other related documentation given to participants and authorised surrogates | Available on dermand |
| Biological specimens | 33 | Plans for collection, laboratory evaluation, and storage of biological specimens for genetic or molecular analysis in the current trial and for future use in ancillary studies, if applicable | NA |

*It is strongly recommended that this checklist be read in conjunction with the SPIRIT 2013 Explanation & Elaboration for important clarification on the items. Amendments to the protocol should be tracked and dated. The SPIRIT checklist is copyrighted by the SPIRIT Group under the Creative Commons “[Attribution-NonCommercial-NoDerivs 3.0 Unported](http://www.creativecommons.org/licenses/by-nc-nd/3.0/)” license.

**Appendix 1 - Items from the World Health Organization Trial Registration Data Set (in parentheses: protocol page number where item is addressed)**

- **Primary Registry and Trial Identifying Number (p4)**
  Name of Primary Registry, and the unique ID number assigned by the Primary Registry to this trial.
- **Date of Registration in Primary Registry (p4)**
  Date when trial was officially registered in the Primary Registry.
- **Secondary Identifying Numbers (p4)**
  Other identifiers besides the Trial Identifying Number allocated by the Primary Registry, if any. These include:
  - The Universal Trial Number (UTN)
  - Identifiers assigned by the sponsor (record Sponsor name and Sponsor-issued trial number (e.g. protocol number))
  - Other trial registration numbers issued by other Registries (both Primary and Partner Registries in the WHO Registry Network, and other registries)
  - Identifiers issued by funding bodies, collaborative research groups, regulatory authorities, ethics committees / institutional review boards, etc.

All secondary identifiers will have 2 elements: an identifier for the issuing authority (e.g. NCT, ISRCTN, ACTRN) plus a number.

There is no limit to the number of secondary identifiers that can be provided.

- **Source(s) of Monetary or Material Support (p21)**
  Major source(s) of monetary or material support for the trial (e.g. funding agency, foundation, company, institution).
- **Primary Sponsor (available in the CLinicalTrials.org registry; NCT02956694)**
  The individual, organization, group or other legal entity which takes responsibility for initiating, managing and/or financing a study. The Primary Sponsor is responsible for ensuring that the trial is properly registered. The Primary Sponsor may or may not be the main funder.
- **Secondary Sponsor(s) (NA)**
  Additional individuals, organizations or other legal persons, if any, that have agreed with the primary sponsor to take on responsibilities of sponsorship.

  A secondary sponsor may have agreed to:
  - take on all the responsibilities of sponsorship jointly with the primary sponsor; or
  - form a group with the Primary Sponsor in which the responsibilities of sponsorship are allocated among the members of the group; or
  - act as the Primary Sponsor’s legal representative in relation to some or all of the trial sites.
- **Contact for Public Queries (available in the CLinicalTrials.org registry; NCT02956694)**
  Email address, telephone number and postal address of the contact who will respond to general queries, including information about current recruitment status.
- **Contact for Scientific Queries (p.2)**
  There must be clearly assigned responsibility for scientific leadership to a named Principal Investigator. The PI may delegate responsibility for dealing with scientific enquiries to a scientific contact for the trial. This scientific contact will be listed in addition to the PI.

  The contact for scientific queries must therefore include:
  - Name and title, email address, telephone number, postal address and affiliation of the Principal Investigator, and;
  - Email address, telephone number, postal address and affiliation of the contact for scientific queries about the trial (if applicable). The details for the scientific contact may be generic (that is, there does not need to be a named individual): e.g. a generic email address for research team members qualified to answer scientific queries.
- **Public Title (available in the CLinicalTrials.org registry; NCT02956694)**
  Title intended for the lay public in easily understood language.
- **Scientific Title (p1)**
  Scientific title of the study as it appears in the protocol submitted for funding and ethical review. Include trial acronym if available.
- **Countries of Recruitment (p8)**
  The countries from which participants will be, are intended to be, or have been recruited at the time of registration.
- **Health Condition(s) or Problem(s) Studied (p1: in Title)**
  Primary health condition(s) or problem(s) studied (e.g., depression, breast cancer, medication error).

  If the study is conducted in healthy human volunteers belonging to the target population of the intervention (e.g. preventive or screening interventions), enter the particular health condition(s) or problem(s) being prevented.
- **Intervention(s) (p13-14, Figure 3)**
  For each arm of the trial record a brief intervention name plus an intervention description.

  Intervention Name: For drugs use generic name; for other types of interventions provide a brief descriptive name.
  - For investigational new drugs that do not yet have a generic name, a chemical name, company code or serial number may be used on a temporary basis. As soon as the generic name has been established, update the associated registered records accordingly.
  - For non-drug intervention types, provide an intervention name with sufficient detail so that it can be distinguished from other similar interventions.

Intervention Description: Must be sufficiently detailed for it to be possible to distinguish between the arms of a study (e.g. comparison of different dosages of drug) and/or among similar interventions (e.g. comparison of multiple implantable cardiac defibrillators). For example, interventions involving drugs may include dosage form, dosage, frequency and duration.

If the intervention is one or more drugs then use the International Non-Proprietary Name for each drug if possible (not brand/trade names). For an unregistered drug, the generic name, chemical name, or company serial number is acceptable.

If the intervention consists of several separate treatments, list them all in one line separated by commas (e.g. "low-fat diet, exercise").

For controlled trials, the identity of the control arm should be clear. The control intervention(s) is/are the interventions against which the study intervention is evaluated (e.g. placebo, no treatment, active control). If an active control is used, be sure to enter in the name(s) of that intervention, or enter "placebo" or "no treatment" as applicable. For each intervention, describe other intervention details as applicable (dose, duration, mode of administration, etc).

- **Key Inclusion and Exclusion Criteria (p8)**
  Inclusion and exclusion criteria for participant selection, including age and sex. Other selection criteria may relate to clinical diagnosis and co-morbid conditions; exclusion criteria are often used to ensure patient safety.

  If the study is conducted in healthy human volunteers not belonging to the target population (e.g. a preliminary safety study), enter "healthy human volunteer".
- **Study Type (p7, 9, 13)**
  Study type consists of:
  - Type of study (interventional or observational)
  - Study design including:
    - Method of allocation (randomized/non-randomized)
    - Masking (is masking used and, if so, who is masked)
    - Assignment (single arm, parallel, crossover or factorial)
    - Purpose
  - Phase (if applicable)

For randomized trials: the allocation concealment mechanism and sequence generation will be documented.

- **Date of First Enrollment (Figure 4)**
  Anticipated or actual date of enrolment of the first participant.
- **Target Sample Size (p10, 16)**
  Number of participants that this trial plans to enrol in total.
- **Recruitment Status (available in the CLinicalTrials.org registry; NCT02956694)**
  Recruitment status of this trial:
  - Pending: participants are not yet being recruited or enrolled at any site
  - Recruiting: participants are currently being recruited and enrolled
  - Suspended: there is a temporary halt in recruitment and enrolment
  - Complete: participants are no longer being recruited or enrolled
  - Other
- **Primary Outcome(s) (p15)**
  Outcomes are events, variables, or experiences that are measured because it is believed that they may be influenced by the intervention.

  The Primary Outcome should be the outcome used in sample size calculations, or the main outcome(s) used to determine the effects of the intervention(s). Most trials should have only one primary outcome.

  For each primary outcome provide:
  - The name of the outcome (do not use abbreviations)
  - The metric or method of measurement used (be as specific as possible)
  - The timepoint(s) of primary interest

Example:
Outcome Name: Depression
Metric/method of measurement: Beck Depression Score
Timepoint: 18 weeks following end of treatment

- **Key Secondary Outcomes (p14-15, Table 3)**
  Secondary outcomes are outcomes which are of secondary interest or that are measured at timepoints of secondary interest. A secondary outcome may involve the same event, variable, or experience as the primary outcome, but measured at timepoints other than those of primary interest.

  As for primary outcomes, for each secondary outcome provide:
  - The name of the outcome (do not use abbreviations)
  - The metric or method of measurement used (be as specific as possible)
  - The timepoint(s) of interest
